# Poly(lactic Acid) Fibers for Sustained Drug Delivery: Insights into Release Profiles and Cellular Interactions

**DOI:** 10.3390/ma18112532

**Published:** 2025-05-27

**Authors:** Elena Mazzinelli, Marianna Messina, Ilaria Favuzzi, Federica Vincenzoni, Alessia Giannoccolo, Ilaria Cacciotti, Giuseppina Nocca

**Affiliations:** 1 Dipartimento di Scienze Biotecnologiche di Base, Cliniche Intensivologiche e Perioperatorie, Università Cattolica del Sacro Cuore, Largo Francesco Vito 1, 00168 Rome, Italy; elena.mazzinelli@unicatt.it (E.M.); ilaria.favuzzi@gmail.com (I.F.); federica.vincenzoni@unicatt.it (F.V.); giuseppina.nocca@unicatt.it (G.N.); 2 Dipartimento di Ingegneria, INSTM RU, Università degli Studi Niccolò Cusano, via Don Carlo Gnocchi 3, 00166 Rome, Italy; marianna.messina@unicusano.it (M.M.); alessia.giannoccolo@unicusano.it (A.G.); 3 Fondazione Policlinico Universitario A. Gemelli, Istituto di Ricovero e Cura a Carattere Scientifico, Largo Agostino Gemelli 8, 00168 Rome, Italy

**Keywords:** polylactic acid fibers, dexamethasone, clobetasol propionate, drug delivery, fibroblasts, cellular drug uptake

## Abstract

Drug delivery systems have revolutionized the clinical field by enabling the targeted, controlled, and sustained release of therapeutic agents, minimizing side effects and maximizing efficacy. Among the various drug delivery platforms, polymer-based systems have gained significant attention due to their versatility and biocompatibility. This study investigates the release of dexamethasone and clobetasol propionate from PLA (poly(lactic acid)) fibers in a cellular culture system. The release profiles were analyzed over 1, 6, and 24 h using high-performance liquid chromatography (HPLC), showing a gradual, continuous release, with clobetasol exhibiting slower release due to its poor water solubility. The presence of fibroblasts did not significantly affect the drug release, and the concentrations increased over time. An intracellular recovery test revealed that both drugs entered the cells, although their concentrations were below the limit of quantification (LOQ). Measurements of the remaining drug in the fibers confirmed a sustained release, with no significant difference between conditions with and without cells. These results highlight the potential of PLA fibrous membranes for controlled drug delivery, though further research is needed to optimize release and improve intracellular quantification for more effective therapeutic applications.

## 1. Introduction

Topical corticosteroids such as dexamethasone (DEX) and clobetasol propionate (CLOB) are widely used in dermatological practice due to their potent anti-inflammatory [1], immunosuppressive [2], and antiproliferative properties [2,3]. DEX, a synthetic glucocorticoid, is commonly applied in the treatment of inflammatory skin conditions such as atopic dermatitis, contact dermatitis, and psoriasis [4]. Its efficacy lies in its ability to inhibit the synthesis of pro-inflammatory cytokines and reduce leukocyte migration, thereby alleviating redness, swelling, and itching [5]. Additionally, DEX is sometimes incorporated into formulations designed to manage localized inflammatory responses in conditions like eczema or chronic venous insufficiency ulcers. CLOB, on the other hand, is one of the most potent corticosteroids available for topical use and is reserved for severe dermatoses unresponsive to less potent agents. It is indicated for the short-term management of conditions such as severe psoriasis, lichen planus, and discoid lupus erythematosus, where its strong anti-inflammatory effects are particularly beneficial [6]. Due to its potency, CLOB is typically prescribed for limited durations and on small surface areas to minimize the risk of systemic absorption and adverse effects, such as skin atrophy or hypothalamic–pituitary–adrenal axis suppression [7].

Despite their efficacy, conventional drug administration often exhibits several significant limitations, including low bioavailability, the risk of overdose, and the difficulty of maintaining consistent drug levels over time [8]. In recent decades, the field of biomedical sciences has witnessed significant advancements in the development of new drug delivery technologies designed to improve therapeutic efficacy. Among these, polymeric nanostructured drug delivery systems have emerged as particularly promising tools [9]. These systems are engineered to provide controllable, prolonged, or targeted drug release, protecting therapeutic agents from harsh physiological environments while optimizing local drug concentrations.

One of the most interesting and innovative strategies is the use of nanostructured materials, particularly electrospun fibers [8]. These fibrous structures, produced using the electrospinning technique, have demonstrated exceptional potential as vehicles for the controlled release of drugs and active ingredients, due to their high specific surface area, controllable porosity, and the ability to encapsulate drugs [10,11]. The integration of electrospun fibers into drug delivery systems represents a promising frontier in biomedical medicine to address therapeutic challenges. The high specific surface area and encapsulation capacity of electrospun fibers enable the controlled and targeted release of drugs, thereby enhancing therapeutic efficacy and reducing side effects [8]. Encapsulation can be achieved either through the inclusion of the drug during the electrospinning process or via subsequent surface coatings. At the same time, the unique properties of the fibers, such as biodegradability and the ability to mimic the extracellular matrix, open new possibilities for tissue regeneration applications and the development of personalized therapies [8].

In this framework, the present study focused on the preparation and physicochemical characterization of polylactic acid (PLA) fibers loaded with corticosteroids, in order to evaluate the potentiality of using fibrous membranes as promising and efficient delivery systems for improving and maximizing the corticosteroids’ therapeutic benefits while simultaneously reducing their side effects. PLA was selected as a polymeric matrix for the incorporation of topical corticosteroids due to its biocompatibility, biodegradability, and non-toxicity, making it suitable for both structural and functional biomedical applications [12,13]. Indeed, from a biomedical perspective, PLA offers additional advantages: it is FDA-approved, well tolerated in human tissues, and can be processed into highly nanostructured forms, such as electrospun fibers, with the goal of creating innovative and biocompatible release systems. Thanks to its versatility, PLA is therefore considered an ideal material for the development of advanced drug delivery systems, particularly when aiming to achieve prolonged, localized, and controlled release. The novelty of this system lies in the integration of corticosteroid drugs within a biodegradable fibrous matrix, capable of releasing the active ingredient in a controlled and targeted manner. This strategy maximizes the therapeutic efficacy of topical treatments while minimizing the systemic side effects typically associated with conventional administration. By leveraging advanced delivery systems, this study aimed to evaluate the release capability of these fibers, quantifying the amount of drug delivered into the culture medium and within cells.

While several studies have already investigated the incorporation of DEX into electrospun delivery systems [14], no systematic research to date has focused on the encapsulation and controlled topical delivery of CLOB using nanofibrous matrices [14,15]. Considering the high pharmacological potency and risk profile of CLOB, its integration into a highly controllable delivery platform is particularly relevant. The innovative aspect of this work lies in the application of the same electrospun PLA fiber-based delivery system to both DEX—a corticosteroid already explored in nanostructured systems [14]—and, for the first time, CLOB. Although the two corticosteroids were not co-loaded within the same membrane, the study explores their behavior within the same type of nanofibrous platform. This dual evaluation aims to determine whether a common polymeric matrix can optimize the release profiles of corticosteroids with different potencies and clinical indications, potentially offering a versatile and safer alternative to conventional formulations.

## 2. Materials and Methods

### 2.1. Materials

Unless otherwise indicated, all materials used in this work were obtained from Sigma Aldrich (Milan, Italy) and used as received. PLA (3051D; density of 1.25 g/cm^3^, average molecular weight numerical (Mn) of approx. 1.42 × 10^4^ g/mol) was purchased from Nature Work (Minneapolis, MN, USA), chloroform (CHCl_3_, 99%), N-Dimethylformamide (DMF; (CH_3_)_2_NOCH, 99.8%), and acetic acid (C_2_H_4_O_2_) were obtained from VWR International. Acetonitrile (CH_3_CN, HPLC-grade; VWR chemical, Milan, Italy) and ultrapure water (obtained by a P.Nix Power I System apparatus; Human Corporation, Seoul, Republic of Korea) were used for high-performance liquid chromatography (HPLC) analysis.

#### Cell Cultures

The 3T3-Swiss mouse fibroblasts (kindly provided by Prof Giovan Battista Pani, UCSC) were cultured at 37° C in a humidified atmosphere with 5% CO_2_, in DMEM supplemented with 10% FBS, penicillin (100 units/mL), streptomycin (0.1 mg/mL), and L-glutamine (2 mmol/L).

### 2.2. Methods

#### 2.2.1. Preliminary Studies with Free-Form Drugs

##### Evaluation of Cytotoxicity Induced by DEX and CLOB

3-(4,5-Dimethylthiazol-2-yl)-2,5-diphenyltetrazolium bromide (MTT) tests were conducted using DEX and CLOB in their free form to determine the non-cytotoxic concentration for subsequent conveyance. For this purpose, 3T3-Swiss fibroblasts were treated with decreasing concentrations of the drugs. Stock solutions were freshly prepared before use, with the drugs dissolved in DMSO at various concentrations.

The final concentrations of the drugs were as follows:

DEX: 0.100 µg/µL, 0.050 µg/µL, 0.025 µg/µL, 0.012 μg/µL;
CLOB: 0.100 μg/µL, 0.050 μg/µL, 0.025 μg/µL, 0.12 μg/µL.

The final concentration of DMSO, used as the vehicle, remained consistent in all samples during the experiments (0.1% *v*/*v*).

The 3T3-Swiss fibroblasts were plated in a 96-well tissue culture dish at a density of 1 × 10^4^ cells/well in DMEM. After 24 h of incubation, the medium was removed, and the cell monolayer was incubated with the drug at the final concentrations mentioned above. After another 24 h of incubation, cell viability was assessed using the MTT assay, as described by Wataha J. et al. [16]. In brief, 100 µL of an MTT solution in PBS (5 mg/mL) was added to each well, and after a 3 h incubation at 37 °C, the produced intracellular formazan crystals were solubilized with an isopropanol solution containing HCl (4 × 10^−2^ M, 0.20 mL). The absorbance of the solutions in each well was measured using an automatic microplate photometer (ELx800; BioTek, Bad Friedrichshall, Germany) at a wavelength of 562 nm. Each experiment was performed in sextuplicate and repeated three times. Cytotoxicity was calculated using Equation (1) [17].

% cell mortality = (Abs ctrl − Abs treated)/(Abs ctrl) × 100(1)

Equation (1): cellular mortality evaluation.

Specimens were classified as slightly, moderately, or severely cytotoxic when the toxic effects, relative to controls, were <30%, between 30% and 60%, or >60%, respectively [18].

##### Chromatographic Conditions

The determination of the chromatographic conditions of the two active ingredients was carried out through high-performance/pressure liquid chromatography (HPLC) analysis to evaluate the concentration of the drugs. Analyses were conducted utilizing an HPLC system, comprising a Thermo Finnigan MS pump, a photodiode array (PDA) detector, and an autosampler. A C-18 (3 µm) Supelco reversed-phase column measuring 150 × 4.7 mm was employed for chromatographic separation. For CLOB, a modified method based on the procedure reported by Pawar et al. [19] was used. In the case of DEX, the chromatographic method was developed and characterized by our research group, based on the conditions reported by [20].

For all analyses, a flow rate of 300 μL/min and an injection volume of 100 μL were set.

For CLOB, a mobile phase composed of 70% acetonitrile and 30% water was used, with detection carried out at a wavelength of 241 nm. For DEX, a mobile phase consisting of 50% acetonitrile and 50% water was selected, and all analyses were performed at a λ of 254 nm. Both analyses were conducted in isocratic mode.

Calibration curves were constructed by diluting the active ingredients in CH_3_OH at various concentrations ranging from 0.625 to 10 µg/mL for DEX and from 1.5 to 40 μg/mL for CLOB.

The absorbance of each concentration (expressed as area under the curve–AUC) was determined and used during the following experiments to determine the concentration of the drugs in the culture medium and in the cells.

The limit of quantitation (LOQ) and limit of detection (LOD), according to the ICH recommendations [21,22] were determined based on standard deviation of the y-intercept and the slope of the calibration curve (n = 5). Calibration curves were constructed from DEX and CLOB as already reported. The following equations were used for calculating LOD and LOQ:

LOD = (3.3 × SD/slope)

LOQ = (10 × SD/slope)

##### Evaluation of Drug Recovery Efficiency from Cell Lysate

Cells were seeded into flasks and allowed to grow until confluence. After removing the culture medium and performing three washes in PBS, the cells were lysed by freeze–thaw cycles. Then, 3 mL of CH_3_OH was added to the cell lysate, which was divided into three different Eppendorf tubes, each containing 1 mL. One Eppendorf tube was used as a control, 5 μg/mL of DEX was added to the second tube, and 20 μg/mL of CLOB was added to the third tube. All samples were centrifuged at 14,000 rpm for 15 min at 4 °C, sonicated for 2 min, and finally, the supernatant was extracted and subjected to HPLC analysis according to the previously described chromatographic methods. The experiment was performed twice [20].

##### Evaluation of Drug Recovery Efficiency from DMEM

Two different concentrations of both drugs were prepared in DMEM: DEX at 1.0 μg/mL and 2.5 μg/mL, and CLOB at 6.25 μg/mL and 12.5 μg/mL. Each test was run twice. After being frozen overnight at −80 °C, the prepared samples were lyophilized using a VirTis bench-top freeze dryer (6 K; Gardiner, NY, USA). Each sample was filled with 1 mL of H_2_O or CH_3_OH in order to reconstitute the drug’s concentration. The mixture was centrifuged for three minutes at 10,000 rpm in an Eppendorf Mini Spin. The samples were subjected to the chromatographic conditions mentioned above for HPLC analysis [21].

The evaluation of drug recovery efficiency was performed considering the limited solubility of the CLOB in polar solvent, like water. According to the Merck Index [23], the concentration of CLOB and DEX were maintained within the range of their solubility,

#### 2.2.2. Studies of Drugs Vehiculated Through Fibers

##### Production and Characterization of Polylactic Acid Fibers (PLA-FBs)

PLA-FBs, both neat and loaded with DEX or CLOB, were fabricated using electrospinning [15,16]. PLA pellets (15% *w*/*v*) were dissolved in a chloroform–DMF mixture (67:33, in volume) under constant magnetic stirring at 40–50 °C for 2 h to ensure complete dissolution. After cooling to room temperature, either DEX or CLOB (at 1% or 5% wt. relative to PLA) was added, and the solution was stirred overnight at room temperature before electrospinning. In this way, four drug-loaded solutions were prepared, and a control solution without drugs was also produced.

The prepared solution was placed into a glass syringe with an 18 G needle and loaded onto an infusion pump (KDScientific, Holliston, MA, USA). The following electrospinning parameters were set: voltage 12 kV (Spellman model SLM50P300, Hauppauge, New York, NY, USA), needle-collector distance 15 cm, and flow rate 0.5 mL/h. Neat PLA-FBs were produced using the same procedure for reference. After deposition, the membranes (PLA-FBs) were detached from the collector and dried for 24 h.

The morphology of both neat and drug-loaded PLA-FBs was examined using scanning electron microscopy (SEM, Zeiss SUPRA 25, Oberkochen, Baden-Württemberg, Germany). The fibers were mounted on aluminum stubs using conductive carbon adhesive disks and coated with a 50 nm gold film using a sputter coater (Agar Scientific B7234 Agar Scientific, Rotherham, UK) at a density of 19.30 g/cm^3^ and 40 mA/s. Approximately 100 fiber diameters were analyzed using ImageJ software (v1.53) to estimate the average diameter and dispersion. Histograms with corresponding cumulative curves were generated using Origin software. The functional chemical groups of the obtained fibrous mats were identified by acquiring infrared spectra. In particular, the infrared spectroscopy measurements were carried out on both uncoated PLA fibers and PLA fibers coated with chitosan, using the FT-IR Nicolet iS5 instrument equipped with an ATR cell (iD7) under the following conditions: region 500–4000 cm^−1^, number of scans 16, spectral resolution 4 cm^−1^. The thermal properties were studied by differential scanning calorimetry (DSC Q2000). DSC measurements were performed under the following conditions: sample weight ∼ 5 mg, nitrogen atmosphere, range temperature from 0 °C to 300 °C, heating and cooling rates of 10 °C/min. Melting temperatures (T_m_), glass transition temperatures (T_g_), cold crystallization temperatures (T_cc_), cold crystallization enthalpies (ΔH_cc_), melting enthalpies (ΔH_m_) and crystallinity degrees (χ) were evaluated in the first and second heating scans.

The crystallinity degree (χ) was calculated as follows:

$$\chi \%=\frac{\Delta H_{m}-\Delta H_{cc}}{\Delta H_{0,m}\left(1-m_{f}\right)}\cdot 100$$

where ΔH_m_, ΔH_0,m_ and ΔH_cc_ represent scan-related melting enthalpy, melting enthalpy for 100% crystalline PLA material (i.e., 93 J/g (Riga, Zhang, & Collis, 2004)) [24] and cold crystallization enthalpy, respectively; $m_{f}$ is the weight fraction of the filler within the nanohybrid fibers [25].

In order to study the fibers’ mechanical properties, uniaxial tensile tests were performed on dog-bone specimens (width 4.8 mm, length 22.25 mm), at 1.2 mm/min to rupture by an electromechanical machine equipped with a 1 KN load cell (Lonos test), following ASTM D1708 [26] and ASTM D882 [27] for elastic modulus calculation. Five specimens were considered for each electrospun matrix. The ultimate stress (σ_max_) was calculated considering the nominal cross-sectional area of the tensile specimen.

##### In Vitro Release Studies of DEX and CLOB from PLA-FBs

A fixed mass of 2.8 mg of PLA-FBs (corresponding to 140 µg of DEX or CLOB) was placed in 2 mL of ethanol (EtOH) at 37 °C and stirred magnetically at 300 rpm. At predetermined time intervals (30 min, 4 h, 6 h, 24 h), 1 mL of the solution was withdrawn and replaced with 1 mL of fresh solvent (V:V dilution). The amount of drug released was determined by measuring the spectrophotometric absorbance (BECKMAN Coulter DU800, Cassina de Pecchi (Milano), Italy) at a wavelength of 254 nm and comparing it with calibration curves previously obtained within a concentration range of 3 to 100 μg/mL. After immersion in EtOH, the PLA-FBs were dried and observed at SEM to evaluate possible morphological changes.

##### Effect of DEX-PLA-FBs and CLOB-PLA-FBs on Cell Viability

To evaluate the potential toxic effects of PLA-FBs, an MTT assay was conducted on the 3T3-Swiss fibroblasts. For this test, cells were seeded in a 12-well plate at a density of approximately 2 × 10⁴ cells per well in a final volume of 2 mL of DMEM. After 24 h of incubation, when the cells reached sub-confluence and adhered to the surface, the PLA-FBs (2.8 mg) were placed in the wells with the following configurations:

Unloaded PLA-FBs;

PLA-FBs loaded with 5% DEX (70 µg/mL drug concentration);
PLA-FBs loaded with 5% CLOB (70 µg/mL drug concentration);
PLA-FBs loaded with 1% DEX (14 µg/mL drug concentration);
PLA-FBs loaded with 1% CLOB (14 µg/mL drug concentration).

Each concentration was tested in duplicate, and two additional replicates were used as controls. The experiment was repeated twice. The plate was incubated for another 24 h, after which 200 μL of MTT solution was added to each well. The plate was incubated at 37 °C for 3 h. Once the reaction was complete, the formazan crystals formed in the viable cells were dissolved using 2 mL of a 0.04 M acidic isopropanol solution. Finally, the absorbance at 562 nm was measured using an automatic plate reader (BioTek Elx800, Bad Friedrichshall, Germany), and cytotoxicity was calculated according to Equation (1).

##### In Vitro Release Studies of DEX and CLOB from PLA-FBs in DMEM and Intracellular Concentration Determination

The 3T3-Swiss fibroblasts were seeded in 12-well plates with a final volume of 2 mL of DMEM per well. After 24 h of incubation, when cell adhesion and sub-confluence were achieved, the PLA-FBs (loaded with 5% DEX or with 5% CLOB) were added. Each PLA-FBs sample weighed 2.8 mg with an active ingredient concentration of 70 μg/mL. The test was conducted twice, and after 1, 6 and 24 h of incubation, drug release was analyzed for both the PLA-FBs. Briefly, 2 mL of medium were collected after 1 h of incubation, additional 2 mL were collected after 6 h, and final 2 mL after 24 h, frozen at −80 °C, lyophilized, and analyzed by HPLC under the conditions specified in chromatographic conditions’ section. To assess the amount of drug remaining in the PLA-FBs, they were immersed in 2 mL of EtOH and stirred at 300 rpm. After 1 h, 1 mL of the solution was collected and analyzed by HPLC, and an additional 1 mL of ethanol was added to continue monitoring drug release after an additional 5 and 23 h. Finally, the steps outlined in the chromatographic conditions’ section were followed to assess the possibility of quantifying the active ingredients intracellularly. All the steps described were also performed in a cell-free condition, which served as a control.

### 2.3. Statistical Analysis

All results are reported as the mean ± standard deviation (SD), based on at least three separate experiments conducted in duplicate. The means were analyzed using analysis of variance (ANOVA), and if significant differences were found, a multiple comparison of means was performed using the Student–Newman–Keuls test. The significance level was set at 0.05.

## 3. Results

### 3.1. Evaluation of Cytotoxicity Induced by DEX and CLOB Without a Carrier

**Figure 1:**
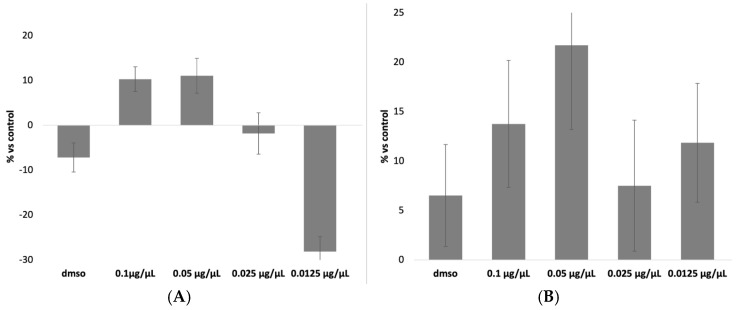
Cell mortality induced by different concentrations of free DEX (**A**) and CLOB (**B**). Data reported as mean mortality ± SD. No statistically significant differences were observed. *p* > 0.05.

The results of the MTT assay for DEX and CLOB were, as expected, negative for cytotoxic effects (Figure 1). This outcome aligns with the pharmacological characteristics of these corticosteroids, which primarily exert their effects by modulating inflammatory pathways and immune responses rather than compromising cell viability [5,6]. At concentrations approximating therapeutic levels, these compounds are specifically designed to act within a safe therapeutic window, minimizing the risk of cellular damage.

Furthermore, the concentrations employed in the assay were selected to reflect those used in clinical settings and to remain well below the thresholds typically associated with direct cytotoxicity. The negative value observed for the 0.0125 µg/µL concentration indicates that, compared to the control, this concentration did not induce cell mortality but rather showed a slight proliferative effect. Since the results are expressed as a percentage of mortality relative to the control group, a negative value signifies that this concentration was the least toxic, demonstrating no harmful effects on the cells.

Among the tested concentrations, 0.07 µg/µL was identified as the most appropriate for both DEX and CLOB, as it is the closest to the therapeutic dose currently administered in clinical practice. Therefore, this concentration was chosen for subsequent experiments to ensure pharmacological relevance while minimizing the risk of cytotoxic effects unrelated to the carrier system.

### 3.2. Chromatographic Conditions

To be sure of detecting the exact drug concentration in intracellular and in DMEM conditions, both chromatographic methods were first tested for linearity, using calibrating standard solution mixtures of the DEX and CLOB. Each determination was repeated three times and each calibration curve was performed five times (n = 5). In the selected chromatographic condition, the retention time (RT) for DEX was 15.91 min and for CLOB was 19.64 min.

The obtained regression equations are reported in Table 1, and the results obtained for the coefficient of determination (R^2^) in the linearity tests for DEX (0.9915) and CLOB (0.926) demonstrated suitable levels of linearity for the purposes of this study, with differences reflecting the characteristics of the respective analytical methods.

The R^2^ for DEX indicates excellent linearity. This result confirms that the analytical method is highly reliable in describing the relationship between concentration and analytical response, with over 99% of the variability explained by the linear regression. Such a high level of linearity meets the most stringent criteria, ensuring that the method is fully suitable for applications requiring high precision and accuracy, such as pharmacokinetic studies or quality control analyses.

The R^2^ for CLOB, although lower than that for DEX, is still acceptable for the purposes of this study. This level of linearity demonstrates that the method can adequately represent the relationship between concentration and analytical response within the range considered. While it is acknowledged that approximately 7.4% of the variability is not explained by the linear regression, the results are sufficiently robust for the analytical needs of our experimental framework.

In terms of sensitivity, the LOD and LOQ values provide valuable insight into the method’s ability to detect low drug concentrations. The LOD and LOQ values for DEX, which are both lower than those for CLOB, indicate a higher sensitivity for DEX detection. This is particularly important when assessing the pharmacokinetics of DEX in cellular systems, where even very low concentrations may have significant therapeutic or toxicological effects. The high sensitivity ensures that the method can reliably detect DEX at concentrations that are often encountered in biological systems.

For CLOB, the higher LOD and LOQ values (1.8 µg/mL and 5.2 µg/mL, respectively) indicate that the method is less sensitive in comparison to DEX, which is consistent with the lower R^2^ observed. While these values are still suitable for most experimental applications, it may be necessary to optimize the method for greater sensitivity, particularly in cases where precise quantification of CLOB at low concentrations is critical. Further optimization could involve adjustments to the experimental conditions, such as increasing the sensitivity of the detection system or refining the calibration procedure.

Overall, the results obtained for both DEX and CLOB demonstrate that the analytical methods used in this study are effective and reliable for quantifying these corticosteroids within their expected concentration ranges. However, there are notable differences in sensitivity and linearity between the two compounds, which should be taken into account when interpreting the results. The differences observed in the analytical performance of DEX and CLOB underscore the importance of tailoring analytical methods to the specific properties of the compounds under investigation. This study provides a robust framework for future analyses of these drugs, but as with any analytical method, it is essential to consider the limitations and to continuously refine the approach to enhance precision and accuracy.

### 3.3. Efficiency of the Drug Recovery in Cell Lysate and DMEM

The amount of active ingredients recovered from intracellular lysates, using HPLC analysis, was 97% for both the drugs (Table 2).

These results clearly indicate that the intracellular amount of the two drugs is almost completely recovered, suggesting that there are no interactions between the molecules and the cellular contents.

The recovery of DEX and CLOB in DMEM was assessed at two concentrations, both exceeding the LOQ (but always under the limit of the solubility concentration), as summarized in Table 3. Using the analytical method developed in this study, approximately 80% of DEX and 66% of CLOB were successfully recovered and quantified.

These results highlight differences in the chemical nature of the two drugs, which likely influence their solubility and interaction with the DMEM medium. DEX, a relatively hydrophilic corticosteroid, demonstrates higher recovery rates, consistent with its greater solubility in aqueous media such as DMEM. In contrast, CLOB, characterized by its more lipophilic structure, exhibits lower recovery rates, which may be attributed to its reduced solubility and potential adsorption to container surfaces or other hydrophobic interactions in the medium.

Given the inability to achieve complete recovery for both drugs, it will be necessary to evaluate not only the amount released into the medium but also the residual drug content retained within the fibrous carriers during the release experiments. This comprehensive approach will provide a more accurate assessment of the drug release profile and the efficiency of the delivery system.

### 3.4. PLA-FBs Physical Characterization

**Figure 2:**
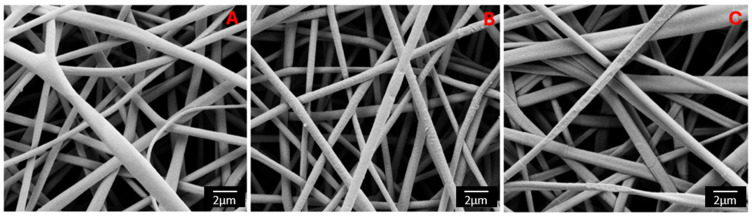
SEM micrographs of unloaded PLA-FBs (**A**), PLA-FBs loaded with DEX (**B**) and PLA-FBs loaded with CLOB (**C**) (magnification 10.00 kx) Scale bar: 2 µm.

The morphology of PLA-FBs, both empty and loaded with DEX or CLOB at two different concentrations (1% and 5%), was observed using SEM. The micrographs shown in Figure 2 depict the different PLA-FBs at the same magnification (Mag = 10.00 kx). It can be observed that there are no surface defects and randomly oriented fibers are obtained, as expected. Precipitates on the surface of PLA-FBs loaded with DEX are evident, attributed to the active ingredient itself.

**Figure 3:**
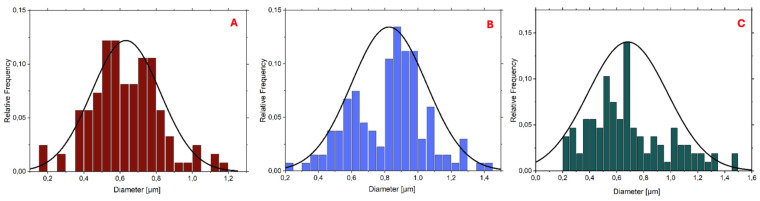
Histogram and cumulative curve of the diameter distributions of PLA-FBs loaded with DEX 5% (**A**), 1%, (**B**) and unloaded (**C**).

**Figure 4:**
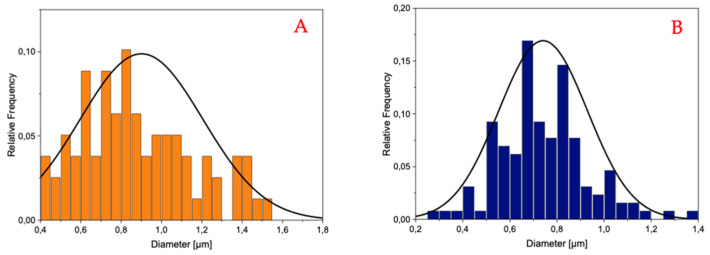
Histogram and cumulative curve of the diameter distributions of PLA-FBs loaded with CLOB 5% (**A**), 1% (**B**).

The histograms reported in Figure 3 and Figure 4 demonstrate that the PLA-FBs typically have diameters ranging between 0.6 and 0.9 μm, on average.

The SEM analysis revealed the presence of two distinct fiber populations, characterized by significantly different diameters within the same electrospun mat. This bimodal distribution suggests the occurrence of independent fiber formation events during the electrospinning process. Several factors may account for this phenomenon, including possible micro-heterogeneities in the polymer solution, local fluctuations in solvent evaporation rates, or the coexistence of different jet instabilities (stable vs. whipping segments) under the same operating conditions. Additionally, in formulations containing dispersed drugs or additives, localized variations in viscosity or conductivity may contribute to the generation of two separate fiber populations. This observation highlights the complexity and sensitivity of the electrospinning process to subtle physicochemical and environmental factors, and suggests that even within optimized systems, multiple fiber formation pathways can coexist [28].

**Figure 5:**
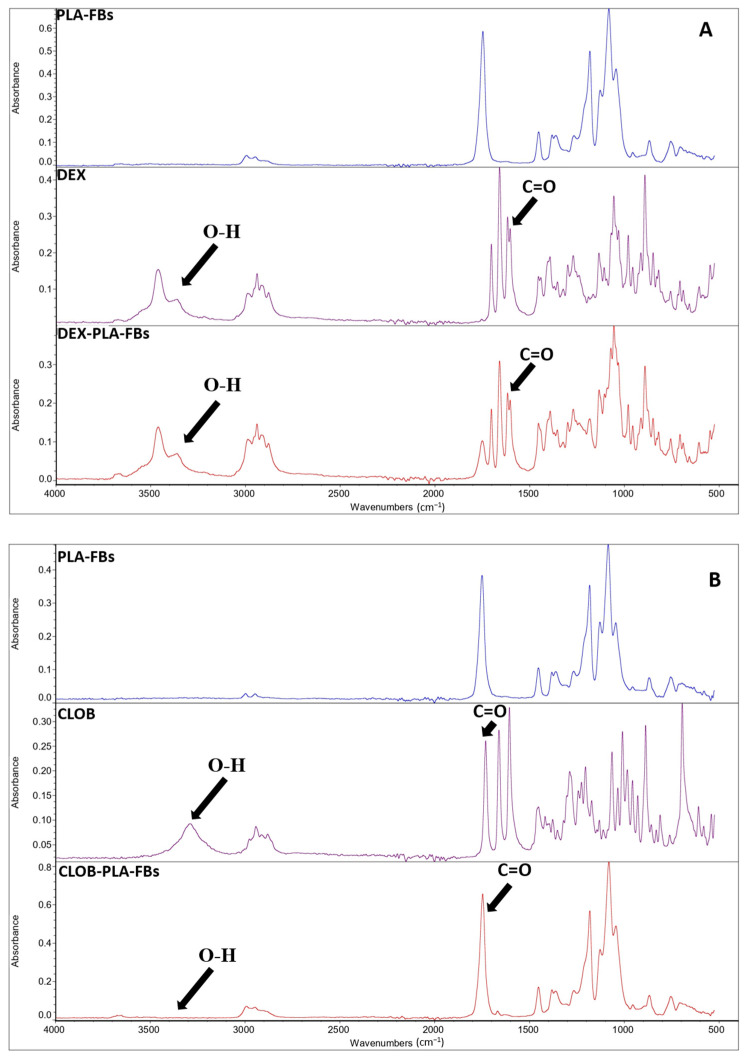
FTIR spectra of: (**A**) DEX (purple curve), PLA-FBs (blue curve), and DEX-PLA-FBs (red curve); (**B**) CLOB (purple curve), PLA-FBs (blue curve), and CLOB-PLA-FBS (red curve).

In order to validate the developed drug delivery systems, it is fundamental to evaluate and confirm the effective drug incorporation and preservation of the molecular structure that are critical prerequisites for subsequent evaluation of the controlled release behavior and biological activity of the fibers. The effective incorporation of the drug within the PLA polymer matrix was confirmed by FTIR measurements, as evident from the reported FTIR spectra of neat PLA fibers, free drugs (dexamethasone and clobetasol), and PLA fibers loaded with 5 wt% of each drug (Figure 5A,B). In detail, in the FTIR spectra of the PLA fibers loaded with 5 wt% of each drug (Figure 5A,B), an additional, albeit less intense, absorption band at around 1660 cm^−1^ is present, in addition to the typical PLA peaks (the main signature of which appears at around 1750 cm^−1^, associated with the ester carbonyl (C=O) group) [20]. This extra band can be ascribed to the stretching vibration of the carbonyl group present in the used corticosteroids (i.e., dexamethasone and clobetasol) [29], confirming the efficacy of the set-up incorporation procedure. The spectroscopic characterization suggests that the drugs are not only physically entrapped within the polymer matrix but also retain their chemical integrity during the incorporation process. Moreover, the absence of significant shifts in the main PLA peaks indicates that no evident chemical reactions occurred between the polymer chains and the active compounds during processing, suggesting a simple physical–mechanical interaction [29].

**Figure 6:**
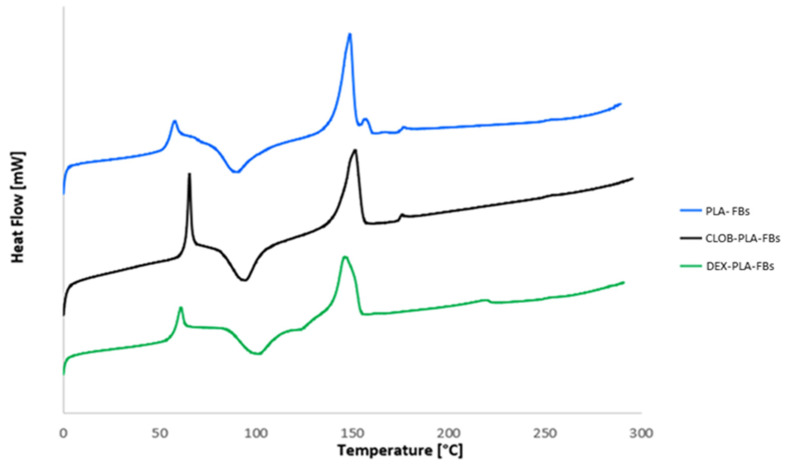
First heating scan DSC thermograms of PLA-based fibers.

DSC analyses were carried out on neat PLA fiber and on PLA fibers loaded with dexamethasone and clobetasol, in order to investigate the influence of the drugs on the thermal properties of the produced fibers. The glass transition (T_g_), melting (T_m_), cold crystallization temperature (T_cc_), melting enthalpies (ΔH_m_), cold crystallization enthalpies (ΔH_cc_) and the crystallinity degree (χ), related to the first and second heating scans, are summarized in Table 4. The first heating scan DSC curves are compared in Figure 6.

From the comparison of the data reported in Table 4, related to the first and second heating process, it was demonstrated that this initial scan highlights both the impact of the drug incorporation and the effect of the electrospinning process on the thermal properties of the PLA matrix (initial comparison figure). Neat PLA fibers exhibited a single melting peak (T_m_) at 149 °C, with a shoulder at a lower temperature, in agreement with other studies [30].

The addition of the drug affected the PLA thermal properties, leading to an increase in both T_g_ and T_cc_, suggesting an interaction between the polymeric chain and the loaded corticosteroid. The increment in glass transition temperature (T_g_) and cold crystallization temperature (T_cc_) suggests that drug incorporation may confer greater rigidity to the polymer matrix, leading to crystallization at higher temperatures. However, all drug-loaded samples exhibited a marked decrease in both melting enthalpy and cold crystallization enthalpy (from 18.62 J/g for neat PLA to approximately 16 J/g for the drug-loaded PLA). This implies that the addition of the drug interferes with the crystallization process, reducing the material’s ability to form crystals at lower temperatures during cooling [29].

As for the degree of crystallinity, it is generally very low in electrospun materials, indicating that most of the polymer chains are in the amorphous state due to the intrinsic characteristics of the electrospinning process (Cacciotti et al., 2014) [31]. In fact, the rapid solvent evaporation and the high elongation rate of the jet during electrospinning usually lead to rapid solidification of the stretched chains in the final stages of the process, hindering the development of crystallinity. Consequently, the chains do not have sufficient time to organize into crystalline structures before solidifying [32]. Comparing the recorded crystallinity degrees, lower values were obtained for the drug-loaded samples, which could indicate that the drug affects the behavior of the polymer chains, preventing them from arranging into a more ordered structure, in agreement with the increased T_cc_.

**Figure 7:**
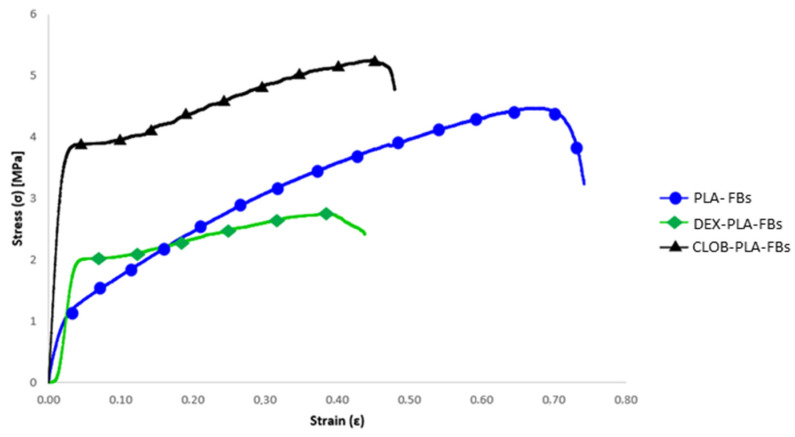
Stress–strain curves of neat and drug-loaded PLA mats.

Concerning the mechanical behaviour, the stress–strain curves of the electrospun mats made of neat PLA and those loaded with drugs (dexamethasone and clobetasol) are compared in Figure 7, and the related data (i.e., maximum tensile strength (σ_max_), yield stress (σ_y_), Young’s modulus (E), and maximum elongation) are summarized in Table 5.

The results obtained from neat PLA samples and drug-loaded samples show significant differences in terms of mechanical performance, as evidenced by the values of maximum tensile strength (σmax), yield stress (σy), Young’s modulus (E), and maximum elongation. In particular, the neat PLA sample showed an average maximum tensile strength of 4.48 MPa, a yield stress of 1.01 MPa, a Young’s modulus of 60 MPa, and a maximum elongation of 70%. These results confirm the good mechanical performance of PLA, known for its relatively high tensile strength and its ability to deform without breaking (Alves et al., 2019) [29].

The PLA sample containing 5% dexamethasone showed a marked reduction in maximum tensile strength (2.76 MPa), but a notable increase in yield stress (1.92 MPa), suggesting that DEX may have a positive effect on initial plastic deformation, while reducing the overall strength of the material. The Young’s modulus decreased to 42 MPa, indicating lower stiffness, while the maximum elongation dropped significantly to 43%, pointing to a less elastic behavior compared to the other samples. On the other hand, the PLA sample containing 5% clobetasol (CLOB) showed the best performance among all tested materials, with a significant increase in maximum tensile strength (5.29 MPa) and yield stress (3.56 MPa), accompanied by a substantial rise in Young’s modulus (188.89 MPa) and a reduced elongation at break, as expected.

As can be seen from the comparative stress–strain curves, the maximum elongation is significantly reduced in the drug-loaded samples, in agreement with the findings reported by [33]. This behavior can be attributed to the interaction between the drug and the polymer matrix, which limits the fibers’ ability to undergo plastic deformation before breaking. Moreover, it is correlated with the DSC results, which evidence increased Tg values (Table 4) for drug-loaded fibers with respect to the neat ones.

### 3.5. In Vitro Release Studies of DEX and CLOB from PLA- FBs in Ethanol

**Figure 8:**
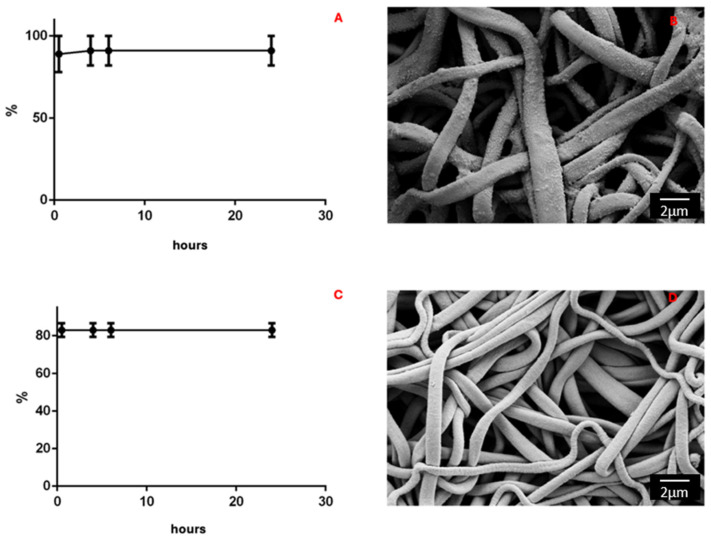
(**A**) DEX release in EtOH in 1 h; (**B**) PLA-FBs with DEX observed though SEM after release in EtOH; (**C**) CLOB release in EtOH in 1 h; (**D**) PLA-FBs with CLOB observed though SEM after release in EtOH. Magnification 10.00 KX. Scale bar: 2 µm.

The drug release in EtOH from PLA-FBs loaded with 5% DEX was 95% in the first hour and no increase was found in the following hours, determining a plateau as shown in Figure 8A. The same trend was observed for the release of CLOB from PLA-FBs; in fact, these fibers were able to release 91.8% of the active ingredient (Figure 8C). Thanks to the SEM analysis, it was possible to observe the deposition of precipitates on the surface of the fibers, attributable to the precipitation of the DEX solubilized in the solvent, following drying (Figure 8B). As for the fibers loaded with CLOB, only a minimal amount of precipitate is observable in the SEM. (Figure 8D).

### 3.6. Effect of DEX and CLOB PLA-FBs on Cell Viability

**Figure 9:**
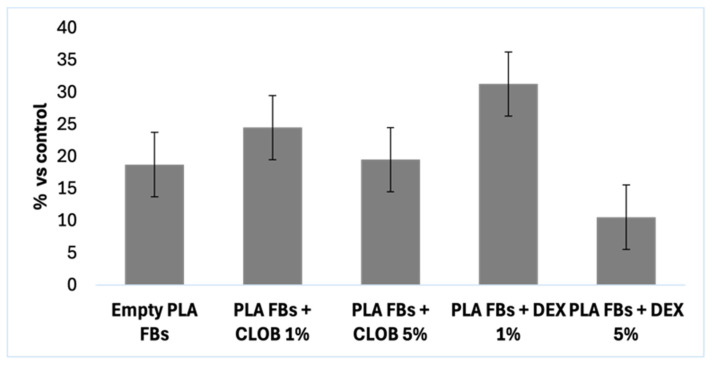
Cell mortality induced by PLA-FBs with 1 and 5% of DEX and CLOB. Data reported as mean mortality ± SD. No statistically significant differences were observed. *p* > 0.05.

Figure 9 shows the results of the cytotoxicity assay conducted on 3T3-Swiss fibroblasts treated with DEX and CLOB delivered via different PLA-FBs, with 1% and 5% drug loading. The cytotoxicity of the unloaded PLA-FBs was also assessed to evaluate the biocompatibility of the polymeric matrix itself. A slight toxic effect was observed for the unloaded PLA-FBs, leading to approximately 10–20% cell mortality. This mild effect is likely related to the polymeric matrix itself, which may have caused minor cellular stress, but it did not significantly compromise cell viability.

Interestingly, the samples treated with drug-loaded fibers showed similar or slightly higher cell viability compared to the unloaded PLA-FBs, indicating that the presence of the active pharmaceutical ingredients did not worsen the cytotoxicity. Notably, PLA-FBs containing 1% DEX exhibited slightly higher cell mortality compared to other formulations, including the 5% DEX-loaded fibers (also if no significant statical differences were observed). This result suggests that the observed toxicity with the 1% DEX-loaded fibers is not solely due to the drug concentration, but could be related to other factors such as the interaction between the polymer matrix and the drug, or the specific characteristics of the drug’s release profile in the cellular environment.

Nevertheless, the maximum cell mortality observed (approximately 30%) still falls within the range generally considered acceptable for in vitro cytocompatibility, as cytotoxic effects below 30% are typically classified as slight and tolerated in cell-based assays [18].

### 3.7. In Vitro Release Capability of PLA-FBs in DMEM and Determination of Intracellular Drugs’ Concentrations

The release of DEX and CLOB from PLA-FBs into the culture medium was evaluated by analyzing DMEM samples taken after 1, 6, and 24 h of incubation, using HPLC. Table 6 shows the results obtained by comparing the AUC values of the samples with the previously established calibration curves. The presence of cells did not affect the drug concentrations in the medium, and the measured concentrations increased over time, demonstrating a continuous release of the drugs from the PLA-FBs.

**Figure 10:**
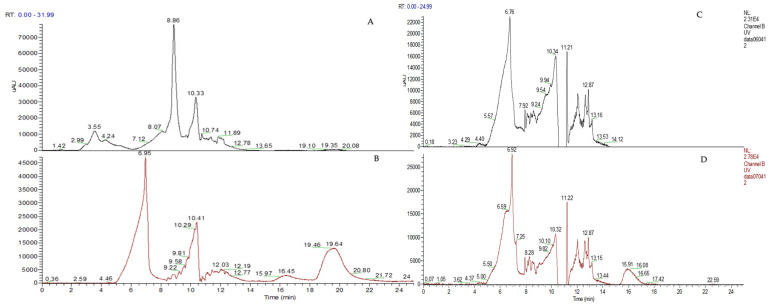
Chromatograms of cellular lysates: (**A**) cellular lysate obtained after incubation of the cells with empty PLA-FBs (control), (**B**) cellular lysate obtained after incubation of the cells with CLOB loaded PLA-FBs, (**C**) cellular lysate obtained after incubation of the cells with empty PLA-FBs (control), (**D**) cellular lysate obtained after incubation of the cells with DEX loaded PLA-FBs. Chromatographic conditions are reported in materials and methods. CLOB RT = 19.64 min; DEX RT: 15.91 min.

An intracellular recovery test was also performed, but the chromatographic results showed concentrations below the LOD. Chromatograms of the intracellular concentrations of DEX and CLOB after 24 h of incubation of PLA-FBs with cells are shown as examples, compared with the controls (Figure 10). These results indicate that the active ingredients can enter the cells, although they cannot be quantified. Moreover, the absence of chromatographic signals at the retention times corresponding to DEX and CLOB in the chromatograms of untreated cell lysates implicitly confirms the absence of endogenous compounds or other interfering substances eluting at those specific retention times. This finding ensures that no coincident signals could compromise the specificity and reliability of the analytical method, thus confirming the selectivity of the assay for the detection and quantification of the two corticosteroids within the cellular matrix.

To account for the incomplete recovery of the active ingredients in the incubation medium, measurements of the remaining active ingredients in the PLA-FBs after 1, 6, and 24 h were also performed. The concentrations observed were similar in both conditions, i.e., with and without cells, indicating that fibroblasts do not affect the release of the active ingredients. Therefore, the obtained data shown in Table 7 demonstrate that the release was gradual for both DEX and CLOB, with the latter showing a slower release due to its poor water solubility.

## 4. Conclusions

The present study was designed to develop and characterize an innovative drug delivery system based on electrospun polylactic acid (PLA) fibers (PLA-FBs) for the topical administration of corticosteroids. The core objective was to explore the feasibility of using the same delivery system for two different active pharmaceutical ingredients—DEX and CLOB—a strategy that represents a novel approach particularly for CLOB, for which no previous studies have employed this type of nanostructured carrier.

Electrospun PLA fibers were selected as the matrix material due to their biocompatibility, biodegradability, and ability to encapsulate and release drugs in a controlled manner. The fibers were successfully loaded with different concentrations (1% and 5% *w*/*v*) of the two corticosteroids, and their physicochemical characteristics, release profiles, cytotoxicity, and intracellular uptake were systematically evaluated.

The in vitro release studies demonstrated a gradual and sustained release of both DEX and CLOB from the PLA-FBs, with release dynamics influenced by the drug’s physicochemical properties. As expected, CLOB exhibited a slower release profile due to its lower water solubility, while DEX showed a more rapid release into the culture medium. Importantly, the presence of fibroblasts did not significantly alter the release behavior of either drug, confirming the system’s stability in a biological environment.

Analytical methods for both corticosteroids were partially characterized to verify linearity, sensitivity, and reliability in quantifying the drugs in both extracellular and intracellular environments. The method for DEX displayed excellent linearity (R^2^ = 0.9915), fully suitable for rigorous quantitative applications, while the method for CLOB, though exhibiting slightly lower linearity (R^2^ = 0.926), was nonetheless considered appropriate for the study’s aims. The intracellular recovery tests confirmed the cellular uptake of both active ingredients, although quantification was limited by the low concentrations detected.

The cytotoxicity assays conducted on 3T3-Swiss fibroblasts revealed that unloaded PLA-FBs exhibited only a mild cytotoxic effect (10–20% cell mortality), consistent with acceptable in vitro biocompatibility thresholds. Notably, the addition of corticosteroids to the fibers did not increase cytotoxicity, and in some cases, drug-loaded fibers demonstrated even higher cell viability than unloaded ones, indicating favorable biocompatibility of the drug-loaded systems.

A relevant point of discussion emerged regarding the fiber diameter distribution within the same electrospinning preparation, where bimodal populations were observed. This phenomenon, unrelated to separate processes, was attributed to local variations in jet formation and polymer droplet instabilities during electrospinning—a well-documented occurrence in the literature, not indicative of process flaws or system inconsistency.

Finally, although drug release profiles were clearly observed over time, no formal kinetic modeling was applied. Future work should therefore aim to optimize release parameters, extend full analytical method validation, apply kinetic release models, and improve intracellular quantification capabilities. These efforts will be crucial to fully harness the potential of PLA-FBs as customizable, biocompatible, and effective carriers for topical corticosteroid therapy, with the added advantage of being adaptable for a range of active ingredients through a single delivery platform.

## Figures and Tables

**Table 1:** Chromatographic results and LOD and LOQ determination.

	DEX	CLOB
Retention time (RT)	15.91 min	19.64 min
Regression equations	Y = 3.292 × 10^6^X + 11437	Y = 2.791 × 10^6^X + 834945
R^2^	0.9915	0.9266
LOD	0.3 µg/mL	1.8 µg/mL
LOQ	0.92 µg/mL	5.2 µg/mL

**Table 2:** CLOB and DEX recovery in cell lysates.

	Drug Added to Cell Lisates (µg/mL)	Recovery Amounts (µg/mL)	Recovery Percentage
**CLOB**	20.00	19.40	97.00%
	20.00	20.10	100.00%
	20.00	19.00	95.00%
**DEX**	5.00	5.10	100.00%
	5.00	4.83	96.60%
	5.00	4.75	95.00%

**Table 3:** Outcomes of the recovery tests for the active ingredients in their unencapsulated form from the DMEM.

	Drug Added to DMEM (µg/mL)	Recovery Amounts (µg/mL)	Recovery Percentage
**CLOB**	12.50	7.50	60.00%
	12.50	8.50	68.00%
	6.25	3.45	55.20%
	6.25	4.12	66.00%
**DEX**	1.00	0.84	84.00%
	1.00	0.89	89.00%
	2.50	1.93	77.00%
	2.50	1.75	70.00%

**Table 4:** DSC data for PLA-based fibrous mats.

**I Heating**						
**Sample**	$T_{g}$ **[°C]**	$T_{m}$ **[°C]**	$T_{cc}$ **[°C]**	ΔHm Jg	ΔHccJg	χ **[%]**
**PLA-FBs**	56	149	90	27.79	18.62	10
**CLOB-PLA-FBs**	64	151	95	20.63	15.79	5
**DEX-PLA-FBs**	60	146	101	20.28	16.95	4
**II Heating**						
**Sample**	$T_{g}$ **[°C]**	$T_{m}$ **[°C]**	$T_{cc}$ **[°C]**	ΔHm Jg	ΔHccJg	χ **[%]**
**PLA-FBs**	58	149	-	22	14	9
**CLOB-PLA-FBs**	65	150	-	16	12	4
**DEX-PLA-FBs**	61	146	-	15	13	2

**Table 5:** Tensile test values for neat and drug-loaded PLA fibers.

Sample	σ_max_ [MPa]	σ_y_ [MPa]	E [MPa]	Maximum Elongation [%]
**PLA-FBs**	4.48 ± 0.1	1.01 ± 0.06	60 ± 7.7	70
**DEX-PLA-FBs**	2.76 ± 0.2	1.92 ± 0.18	43 ± 5.57	43
**CLOB-PLA-FBs**	5.29 ± 0.18	3.56 ± 0.1	189 ± 8.24	46

**Table 6:** DEX and CLOB recovery in DMEM.

	Drug Recovery with Cells			Drug Recovery Without Cells	
	1 h	6 h	24 h	6 h	24 h
**DEX**	10.0 µg/mL	11.9 µg/mL	14.0 µg/mL	8.5 µg/mL	11.6 µg/mL
**CLOB**	1.5 µg/mL	2.2 µg/mL	5.5 µg/mL	2.5 µg/mL	6.0 µg/mL

**Table 7:** DEX and CLOB concentrations into PLA-FBs after incubation in DMEM.

	Drug Recovery with Cells			Drug Recovery Without Cells	
	1 h	6 h	24 h	6 h	24 h
**DEX**	31 µg/mL	28 µg/mL	26 µg/mL	28 µg/mL	26 µg/mL
**CLOB**	61 µg/mL	56 µg/mL	53 µg/mL	57 µg/mL	54 µg/mL

## Data Availability

The original contributions presented in this study are included in the article. Further inquiries can be directed to the corresponding author.
